# Structural Insights into the Molecular Design of Flutolanil Derivatives Targeted for Fumarate Respiration of Parasite Mitochondria

**DOI:** 10.3390/ijms160715287

**Published:** 2015-07-07

**Authors:** Daniel Ken Inaoka, Tomoo Shiba, Dan Sato, Emmanuel Oluwadare Balogun, Tsuyoshi Sasaki, Madoka Nagahama, Masatsugu Oda, Shigeru Matsuoka, Junko Ohmori, Teruki Honma, Masayuki Inoue, Kiyoshi Kita, Shigeharu Harada

**Affiliations:** 1 Department of Biomedical Chemistry, Graduate School of Medicine, The University of Tokyo, Tokyo 113-0033, Japan; E-Mails: danielken@m.u-tokyo.ac.jp (D.K.I.); balogun1@m.u-tokyo.ac.jp (E.O.B.); jnk.ohmori@gmail.com (J.O.); 2 Department of Applied Biology, Graduate School of Science Technology, Kyoto Institute of Technology, Kyoto 606-8585, Japan; E-Mails: tshiba@kit.ac.jp (To.S.); dsato@kit.ac.jp (D.S.); sasaki198603@gmail.com (Ts.S.); qp.m.324@gmail.com (M.N.); 3 Department of Biochemistry, Ahmadu Bello University, Zaria 2222, Nigeria; 4 Research Center, Nihon Nohyaku Co., Ltd., 345 Oyamada-cho, Kawachinagano, Osaka 586-0094, Japan; E-Mail: oda-masatsugu@nichino.co.jp; 5 Department of Integrated Analytical Chemistry, Graduate School of Pharmaceutical Sciences, The University of Tokyo, Tokyo 113-0033, Japan; E-Mails: matsuokas11@chem.sci.osaka-u.ac.jp (S.M.); inoue@mol.f.u-tokyo.ac.jp (M.I.); 6 Center for Life Science Technologies, RIKEN, Tsurumi, Yokohama 230-0045, Japan; E-Mail: honma.teruki@riken.jp

**Keywords:** complex II, NADH-fumarate reductase system, fumarate respiration, *Ascaris suum*, mitochondria, flutolanil, crystal structure, structure-based drug design, antiparasitic agent

## Abstract

Recent studies on the respiratory chain of *Ascaris suum* showed that the mitochondrial NADH-fumarate reductase system composed of complex I, rhodoquinone and complex II plays an important role in the anaerobic energy metabolism of adult *A. suum*. The system is the major pathway of energy metabolism for adaptation to a hypoxic environment not only in parasitic organisms, but also in some types of human cancer cells. Thus, enzymes of the pathway are potential targets for chemotherapy. We found that flutolanil is an excellent inhibitor for *A. suum* complex II (IC_50_ = 0.058 μM) but less effectively inhibits homologous porcine complex II (IC_50_ = 45.9 μM). In order to account for the specificity of flutolanil to *A. suum* complex II from the standpoint of structural biology, we determined the crystal structures of *A. suum* and porcine complex IIs binding flutolanil and its derivative compounds. The structures clearly demonstrated key interactions responsible for its high specificity to *A. suum* complex II and enabled us to find analogue compounds, which surpass flutolanil in both potency and specificity to *A. suum* complex II. Structures of complex IIs binding these compounds will be helpful to accelerate structure-based drug design targeted for complex IIs.

## 1. Introduction

**Scheme 1:**
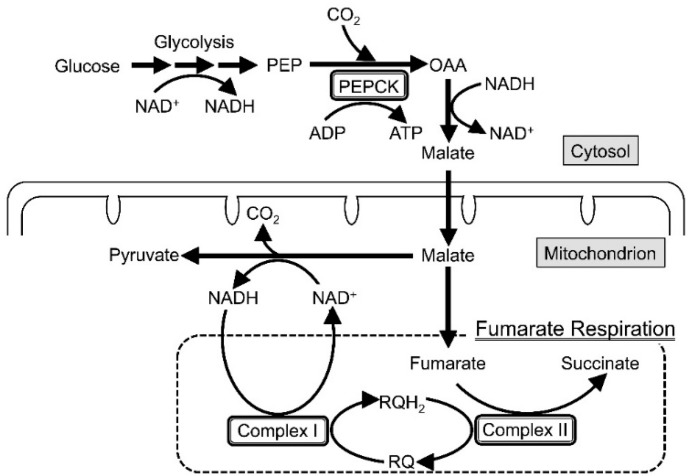
Phosphoenolpyruvate carboxykinase (PEPCK)-succinate pathway of adult *A. suum*. The first step of the pathway converts glucose to fumarate and pyruvate via oxaloacetate and malate. In the second step, fumarate is reduced by the NADH-fumarate reductase system (fumarate respiration), in which electrons are transferred from NADH to fumarate by the action of complexes I and II. PEP: phosphoenolpyruvate; PEPCK: phosphoenolpyruvate carboxykinase; OAA: oxaloacetate; RQ: rhodoquinone; RQH_2_: rhodoquinol.

Recent research on the respiratory chain of adult *Ascaris suum*, a parasitic nematode residing in the hypoxic environment of the host’s small intestine, revealed that ATP synthesis is carried out by the phosphoenolpyruvate carboxykinase (PEPCK)-succinate pathway (Scheme 1) [1,2,3]. In the first step of the pathway, phosphoenolpyruvate (PEP) produced from glycolysis is carboxylated by PEPCK to generate oxaloacetate (OAA), which is then reduced to malate via the oxidation of NADH to NAD^+^ by the reverse reaction of malate dehydrogenase. The produced malate is transported into the mitochondria, where it is converted into pyruvate and fumarate. The second step of the pathway, NADH-fumarate reductase system or fumarate respiration, couples the oxidation of NADH produced by the conversion of malate into pyruvate to the reduction of fumarate to succinate [4].

Fumarate respiration, catalyzed by complexes I and II, is a well known electron transport chain in anaerobic bacteria [4] and also plays an essential role in the regeneration of NAD^+^ that is indispensable for the energy metabolism of adult *A. suum* [3]. The enzymes of the fumarate respiration are anchored in the inner mitochondrial membrane where complex I (NADH-rhodoquinone reductase) acts as a proton pump that is driven by the oxidation of NADH to NAD^+^ and coupled to the reduction of rhodoquinone (RQ) to rhodoquinol (RQH_2_), while complex II, serving as a RQH_2_-fumarate reductase (QFR), transfers electrons from RQH_2_ to fumarate [5]. In contrast, mammalian complex II catalyzes the reverse reaction in mammalian mitochondria, where it acts as succinate-ubiquinone reductase (SQR) in the aerobic respiratory chain. Since fumarate respiration is the major pathway of energy metabolism during adaptation to a hypoxic environment for many species of parasites including adult *A. suum* and bacteria inhabiting anaerobic environments [4,6,7] as well as some human cancer cells exposed to low nutrition and low oxygen conditions [8,9,10,11], the pathway is one of the most promising targets of chemotherapy for both parasitic diseases and cancer [12,13,14]. It has actually been suggested that the target of bithionol and thiabendazole used as drugs for the treatment of paragonimiasis and a fungicide [15,16], respectively, is fumarate respiration, although there is no conclusive evidence.

Hitherto, we have searched for potent inhibitors for *A. suum* QFR in order to identify promising lead compounds for the development of anthelmintics. Since the adult *A. suum* is large in size and known as a representative of human and livestock worms [12,17,18], it is an ideal model parasite for both biochemical analysis and structure-based drug discovery targeted for fumarate respiration [19]. The first compound discovered was Atpenin A5 [20,21,22], but it strongly inhibited both bovine SQR and *A. suum* QFR with IC_50_ values of 0.0036 and 0.012 μM, respectively, and thus could not be developed as a drug. The search continued and finally we found flutolanil, a commercially available fungicide, to be a potent and specific inhibitor for *A. suum* QFR [23], inferred from its IC_50_ values of 0.058 and 45.9 μM for *A. suum* QFR and porcine SQR, respectively. These IC_50_ values account for a selectivity index of approximately 790 folds, and portrays flutolanil to be a promising and safe anthelmintic drug candidate. Here, we describe structures of *A. suum* QFR and porcine SQR in complex with flutolanil determined at 2.91 and 3.0 Å resolution, respectively. These structures show that in both enzymes, flutolanil is bound to their quinone binding sites, and in addition we were able to identify important intermolecular interactions that are responsible for the specificity and potency of flutolanil against *A. suum* QFR. This structural information was proved by the structure-activity relationships of flutolanil derivatives and led to the discovery of novel flutolanil analogues that surpass flutolanil in both inhibitory activity and specificity toward *A. suum* QFR. Importantly, the structures of QFR and SQR in complexes with these analogues would be helpful to advance efforts towards structure-based drug design with flutolanil derivatives.

## 2. Results and Discussion

### 2.1. Structures of Adult Ascaris suum QFR and Porcine Succinate-Ubiquinone Reductase (SQR)

**Figure 1:**
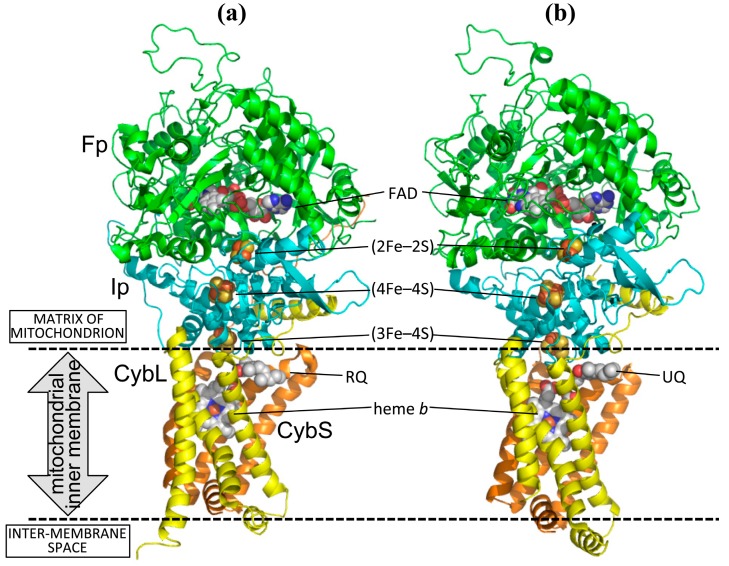
Cartoon representation of structures of (**a**) *A. suum* QFR and (**b**) porcine SQR. Fp (chain A), Ip (chain B), CybL (chain C) and CybS (chain D) subunits are colored in green, cyan, yellow and orange, respectively. Five prosthetic groups, FAD, (2Fe–2S), (4Fe–4S), (3Fe–4S) and heme *b*, as well as bound rhodoquinone (RQ) and ubiquinone (UQ) are shown as spheres with color codes of C (white), N (blue), O (red), S (yellow) and Fe (brown). The identity between amino acid sequences of QFR and SQR is 67%, 62%, 23% and 29% for Fp, Ip, CybL and CybS subunits, respectively. The coordinates of porcine SQR were taken from 1ZOY [26].

**Figure S1:**
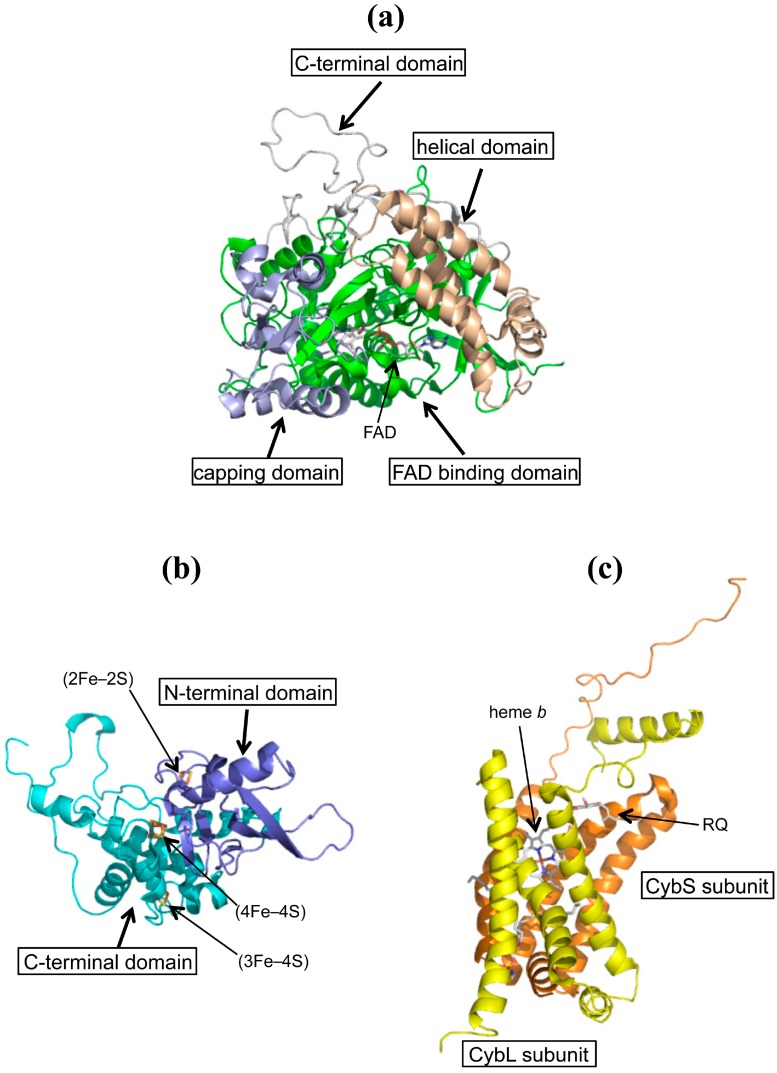
Cartoon representations of (**a**) Fp subunit; (**b**) Ip subunit and (**c**) membrane anchoring CybL/CybS subunits. Domains of Fp and Ip subunits are colored differently. Prosthetic groups are shown as sticks with color codes of C (white), N (blue), O (red), P (orange), S (yellow) and Fe (brown).

**Figure S2:**
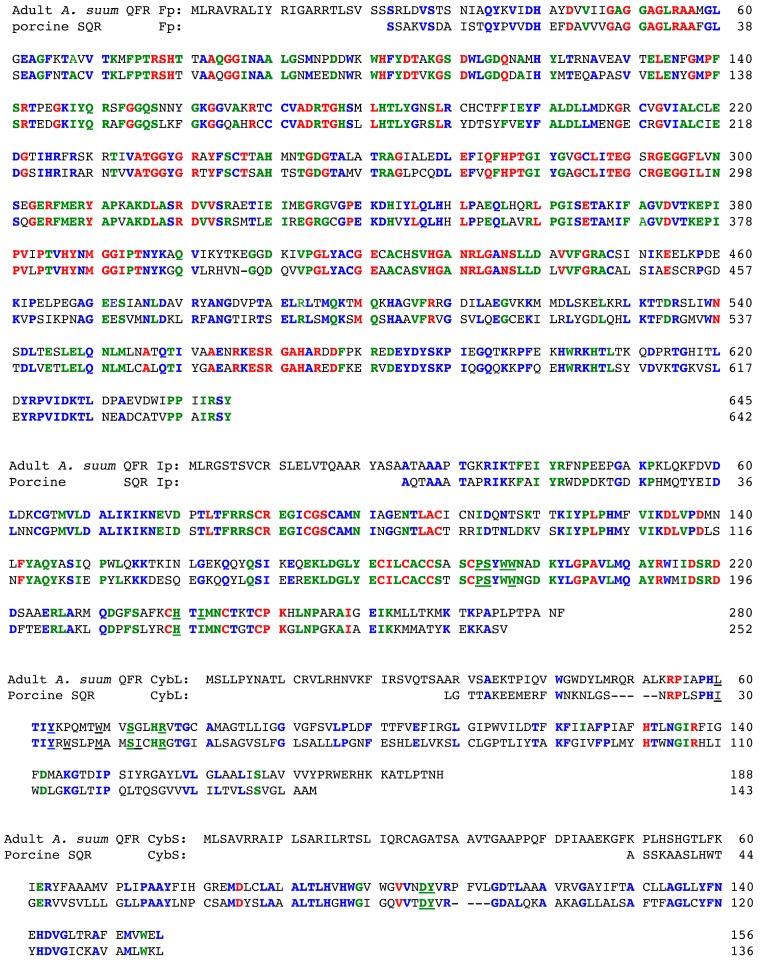
Amino acid sequences of Fp, Ip, CybL and CybS subunits of *A. suum* QFR and porcine SQR. Residues conserved in complex IIs with known structures (SQRs from porcine, chicken an *E. coli*, and QFRs from *E. coli* and *A. suum*) are colored red, residues conserved in SQRs from porcine, chicken, *E. coli* and *A. suum* QFR are colored green, residues conserved in SQRs from porcine, chicken and *A. suum* QFR are colored blue. Residues within 5 Å of the bound quinone are underlined.

Like bacterial and mitochondrial complex IIs with known structures, which includes SQRs from *Escherichia coli* [24], chicken [25] and porcine [26] as well as QFR from *E. coli* [27,28], the structure of *A. suum* QFR [29] is composed of Fp (chain A), Ip (chain B), CybL (chain C) and CybS (chain D) subunits (Figure 1). Notably, the *A. suum* QFR is evolutionally more closely related to the bacterial and mitochondrial SQR (including the larval SQR of *A. suum*) than to bacterial QFR [30,31,32,33]. The Fp subunit contains a FAD binding domain, a capping domain, a helical domain and a C-terminal domain and binds a FAD prosthetic group in the FAD binding domain (Figure S1a). The Ip subunit accommodates three iron-sulfur centers; a (2Fe–2S) iron-sulfur center in an N-terminal plant ferredoxin-like domain, and (4Fe–4S) and (3Fe–4S) centers in a C-terminal bacterial ferredoxin-like domain (Figure S1b). In contrast to the hydrophilic Fp and Ip subunits, the hydrophobic CybL and CybS subunits consist of three membrane-spanning α-helices that anchor *A. suum* QFR to the mitochondrial inner membrane (Figure S1c). The interface between the CybL and CybS subunits binds a heme *b* molecule with coordination bonds from two conserved His 131C and His 95D residues (Figure S2).

**Figure 2:**
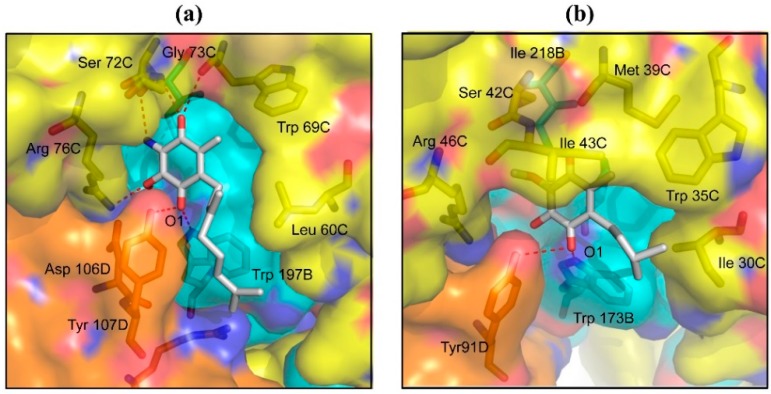
Quinone binding sites and bound quinone molecules of (**a**) *A. suum* QFR and (**b**) porcine SQR (1ZOY). Protein subunits are represented as surface with color codes of Ip (cyan), CybL (yellow) and CybS (orange) and the bound (**a**) RQ and (**b**) UQ are shown as sticks with color codes of C (white), N (blue), O (red). Hydrogen bonds are represented as red dotted lines. Residues represented as sticks are those close to the bound quinone molecules (≤4.0 Å).

The RQ binding site of *A. suum* QFR is constructed by Ip, CybL and CybS subunits and is located in the mitochondrial inner membrane near the surface of the matrix side. Twelve residues (Pro 193B, Ser 194B, Trp 197B, Ile 242B, Leu 60C, Trp 69C, Ser 72C, Gly 73C, Arg 76C, Asp 106D, Tyr 107D, Arg 109D) are close (≤4 Å) to the bound RQ quinone ring (Figure 2a). The UQ binding site of porcine SQR [26] is also formed by equivalent amino acid residues (Figure 2b), of which three (Ile 30C, Met 39C, Ile 43C) are not conserved in *A. suum* QFR (Figure S2). Five residues of *A. suum* QFR (Trp 197B, Ser 72C, Gly 73C, Arg 76C, Tyr 107D) form hydrogen bonds with the quinone ring of the bound RQ (Figure 2a), whereas Ser 42C, Ile 43C and Arg 46C of porcine SQR, the equivalents of Ser 72C, Gly 73C and Arg 76C in *A. suum* QFR do not form hydrogen bonds with the quinone ring of the bound UQ (Figure 2b). This is probably due to the steric hindrance between the bulky side chain of SQR Ile 43C and the bound UQ, obstructing the formation of hydrogen bonds. In *A. suum* QFR, Gly 73C occupies the position of SQR’s Ile 43C (Figure S2). Furthermore, while the quinone rings of the bound RQ and UQ forms hydrogen bonds with residues of quinone binding sites, the isoprene tails extend toward the entrances of the quinone binding sites located in the mitochondrial inner membrane and are not in close contact with amino acid residues.

### 2.2. Structures of Flutolanil Binding Sites of A. suum QFR and Porcine SQR

**Figure 3:**
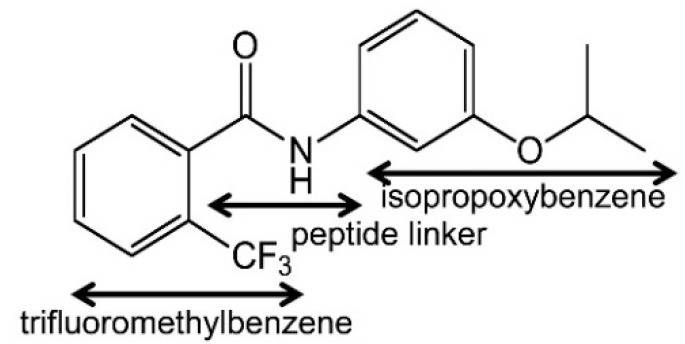
Structure of flutolanil.

**Figure 4:**
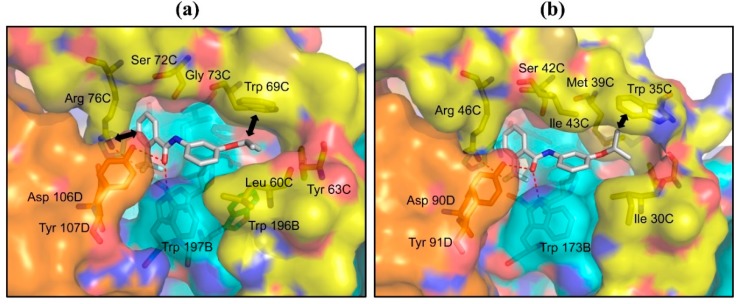
Flutolanil binding sites of (**a**) *A. suum* QFR and (**b**) porcine SQR. Protein subunits are represented as surface with color codes of Ip (cyan), CybL (yellow) and CybS (orange). Flutolanil and residues near to the bound flutolanil (≤4.0 Å) are shown as sticks with color codes of N (blue) and O (red). Carbon atoms of flutolanil and residues of Ip, CybL and CybS are colored white, cyan, yellow and orange, respectively. Hydrogen bonds between flutolanil and residues (*A. suum* QFR: Trp 197B, Arg 76C, Tyr 107D; porcine SQR: Trp 173B, Arg 46C, Tyr 91D) are shown by red dotted lines. Bold bidirectional arrows show: (**a**) the C^δ+^-H…π interaction between the isopropoxy methine group of flutolanil and the indole ring of Trp 69C, and the electrostatic interaction between the trifluoromethylbenzene ring of flutolanil and the Arg 76C guanidino group; and (**b**) the C-H…π interaction between the isopropoxy methyl group and the Trp 69C indole ring.

Flutolanil (*N*-[3-(isopropyloxy)phenyl]-2-(trifluoromethyl)benzamide), a commercially available fungicide [34,35], is made up of two aromatic trifluoromethylbenzene and isopropoxybenzene rings that are connected by a peptide bond linker (Figure 3). This compound is a potent inhibitor for *A. suum* QFR (IC_50_ = 0.058 μM) but poorly inhibits porcine SQR (IC_50_ = 45.9 μM) [14,23]. To obtain insight into its high specificity and potency against *A. suum* QFR, the X-ray structures of *A. suum* QFR and porcine SQR in complex with flutolanil were determined at approximately 2.9 and 3.0 Å resolution, respectively, (Figure 4a,b). The determined structures indicate that in each enzyme, flutolanil is bound to the quinone binding site via hydrogen bonds with three conserved residues (*A. suum* QFR: Trp 197B, Arg 76C, Tyr 107D; porcine SQR: Trp 173B, Arg 46C, Tyr 91D), and there are 13 residues within a distance of 4.0 Å from the bound flutolanil, most of which are conserved in the amino acid sequences of both enzymes (Figure S2). In spite of these similarities, detailed inspection of the structures reveals key interactions that appear to be responsible for the specificity and potency of flutolanil toward *A. suum* QFR.

#### 2.2.1. Interaction between Trifluoromethylbenzene Ring and Arginine Residue

The structure of *A. suum* QFR in complex with flutolanil shows that the trifluoromethylbenzene ring is bound to the same site as the RQ quinone ring and is surrounded mainly by hydrophilic residues (Pro 193B, Ser 194B, Trp 197B, His 240B, Ile 242B, Ser 72C, Arg 76C, Asp 106D, Tyr 107D), of which the guanidino group of Arg 76C interacts with the trifluoromethylbenzene ring of flutolanil via a hydrogen bond and electrostatic interaction (Figure 4a).

Since the binding site of RQ is located in the water-free mitochondrial inner membrane (Figure 1), the guanidino group of Arg 76C would be in the unprotonated electron-rich state due to the lone pair electrons of guanidino nitrogen atoms. On the other hand, the electron-withdrawing property of the trifluoromethyl substituent would induce an uneven distribution of electric charge on the trifluoromethylbenzene ring; while the trifluoromethyl substituent takes on a partial negative charge, the benzene ring becomes an electron-deficient π molecular orbital. The distance between the η1-nitrogen atom of the guanidino group and a fluorine atom of the trifluoromethyl substituent is 3.3 Å, which is shorter than the sum of van der Waals radii of –NH (2.2 Å) and F (1.4 Å), hence, suitable for the formation of a hydrogen bond of >NH…FCF_2_–. In addition, the electron-rich guanidino group of Arg 76C and the electron-deficient benzene ring of the trifluoromethylbenzene ring are disposed face-to-face approximately in parallel at a distance of 3.3 Å (Figure 4a), indicating that they interact with each other via electrostatic interaction. The importance of this electrostatic interaction for the potency and specificity of flutolanil as an inhibitor toward *A. suum* QFR is proved by structure-activity relationship studies with flutolanil derivatives, especially **1** and **2** (Table 1). For *A. suum* QFR, the derivative **1**, which possesses an iodine instead of the trifluoromethyl substituent, shows a slightly high IC_50_ value as compared with flutolanil, but the IC_50_ value of the derivative **2** is considerably higher than that of flutolanil, suggesting that the electrostatic interaction between the guanidino group and the π molecular orbital of the benzene ring is weakened by the electron-donating property of the methyl group.

**Table 1:** Flutolanil derivatives.

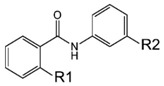

Derivative	R1	R2	IC_50_ (QFR/SQR, μM)	PDB Code (QFR/SQR)
flutolanil	–CF_3_	–O–CH(CH_3_)_2_	0.0581/45.9	5C2T/4YXD
1	–I	–O–CH(CH_3_)_2_	0.0723/6.43	4YSZ/3AE7
2	–CH_3_	–O–CH(CH_3_)_2_	0.515/90.0	4YT0/–
3	–CF_3_	–CH_2_–N(CH_3_)_2_	3.42/256	–/3AEA
4	–CF_3_	–O–C_6_H_5_	0.0794/16.2	–/–
5	–CF_3_	–C_6_H_5_	0.0245/8.61	4YTM/3ABV
6	–CF_3_	–O–C_6_H_5_F_5_	0.330/236	–/3AE9

In the porcine SQR-flutolanil complex (Figure 4b), like *A. suum* QFR, the trifluoromethylbenzene ring is surrounded by a similar set of residues (Pro 169B, Ser 170B, Trp 173B, His 216B, Ser 42C, Arg 46C, Asp 90D, Tyr 91D) and the η1-nitrogen atom of the guanidino group of Arg 46C donates a hydrogen bond to the trifluoromethyl substituent with a distance of 3.2 Å. However IC_50_ values of the derivatives **1** and **2** are comparable with that of flutolanil. This is probably due to the unfavorable arrangement of the guanidino group and the benzene ring for the electrostatic interaction. In fact they poorly overlap with each other and are separated by more than 3.7 Å. This observed difference in the orientation of the interacting groups between the inhibitor and both enzymes explains the importance of the electron-withdrawing property of R1 substituents on the effectiveness of flutolanil as an *A. suum* QFR inhibitor.

#### 2.2.2. C–H…π Interaction between Isopropoxybenzene Ring and Tryptophan Residue

In contrast to the trifluoromethylbenzene, the isopropoxybenzene ring is in a hydrophobic environment constructed mainly by hydrophobic residues (Pro 193B, Trp 196B, Leu 60C, Tyr 63C, Trp 69C, Gly 73C) and is sandwiched between Leu 60C and Trp 69C (Figure 4a). Since, in the structure of flutolanil-free *A. suum* QFR, the side chains of Leu 60C and Trp 69C are in close contact (Figure 2a), it appears that the binding of flutolanil to the enzyme causes separation of the residues so that the isopropoxybenzene ring enters the space between them. Actually, the distance between the two residues increased from 4.4 to 6.3 Å upon binding flutolanil, and the methine group of the isopropoxybenzene forms a C-H...π interaction with the aromatic indole ring of Trp 69C at a distance of 3.3 Å. The separation of the residues might cause energetically unfavorable strain in *A. suum* QFR but it is perhaps compensated for by the interaction between the isopropoxy substituent and Trp 69C.

In the porcine SQR-flutolanil complex, the isopropoxybenzene ring is also surrounded by hydrophobic residues (Pro 169B, Trp 172B, Ile 30C, Tyr 33C, Trp 35C, Met 39C, Ala 40C, Ile 43C) and is inserted into the space between Ile 30C and Trp 35C, which is similarly enlarged from 4.2 to 6.3 Å. However, instead of the isopropoxy methine, the methyl group forms a C–H…π interaction with the indole ring of Trp 35C at a distance of 3.3 Å (Figure 4b). Since the methine group possesses a partial positive charge induced by the isopropoxy oxygen atom, the methine C–H…π interaction is obviously stronger than the methyl C–H…π interaction.

The importance of the partial positive charge on the methine group to the specificity and potency of flutolanil toward *A. suum* QFR is confirmed by the derivative **3** (Table 1), in which a partial negative charge instead of a positive charge is induced on the nitrogen atom of the dimethylaminomethyl group. Probably owing to the repulsion between the partial negative charge on the nitrogen atom and the π molecular orbital of the Trp 69C indole ring, the IC_50_ value of the derivative **3** for *A. suum* QFR drastically increases 59-fold to 3.42 μM from 0.058 μM of flutolanil, whereas for porcine SQR the IC_50_ value increases only 6-fold. Unfortunately, efforts to obtain crystals of the *A. suum* QFR in complex with the derivative **3** are currently unsuccessful, but the structure of the porcine SQR-derivative **3** complex shows that the shortest distance between the nitrogen atom of the derivative **3** and the indole ring of Trp 35C (4.6 Å) becomes longer as compared with the corresponding distance in the porcine SQR-flutolanil complex (4.1 Å), which probably avoids unfavorable interaction between the partially negative nitrogen atom and the indole ring of Trp 35C.

### 2.3. Structures of A. suum QFR and Porcine SQR in Complexes with Flutolanil Derivatives

**Figure 5:**
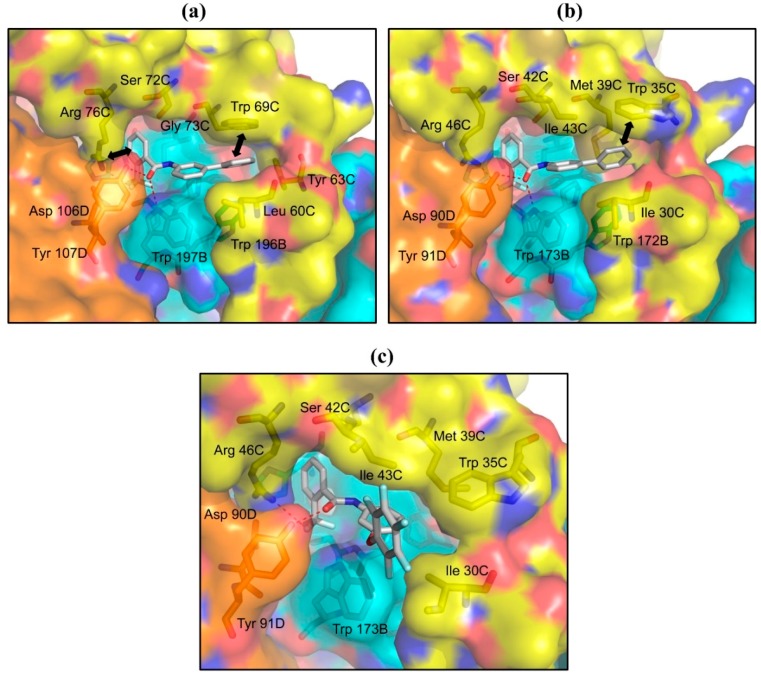
Binding sites of derivative **5** in (**a**) *A. suum* QFR and (**b**) porcine SQR and derivative **6** in (**c**) porcine SQR. Protein subunits are represented as surface, derivatives **5**, **6** and nearby residues (≤4.0 Å) are shown as sticks using the same color codes as Figure 4. Trifluoromethyl fluorine and carbonyl oxygen atoms of derivative **5** form hydrogen bonds (red dotted lines) with residues of *A. suum* QFR (Trp 197B, Arg 76C and Tyr 107D) and with those of porcine SQR (Trp 173B, Arg 46C and Tyr 91D). In addition, as shown by bold bidirectional arrows, derivative **5** forms π–π and electrostatic interactions with Trp 69C and Arg 76C of *A. suum* QFR, respectively, whereas in porcine SQR only π–H…π interaction between Trp 35C and derivative **5** is observed. Trifluoromethyl fluorine and carbonyl oxygen atoms of derivative **6** also accept hydrogen bonds from Trp 173B, Arg 46C and Tyr 91D but do not interact with Trp 35C.

Instead of the isopropoxybenzene group of flutolanil, the R2 groups of the flutolanil derivatives **4**, **5** and **6** are bulky phenoxy, phenyl and pentafluorophenoxy groups, respectively (Table 1). The IC_50_ values of the derivatives **4** and **5** for *A. suum* QFR and porcine SQR are comparable to or even lower than those of flutolanil, but the derivative **6** inhibits both enzymes less effectively. In order to analyze the decreased effectiveness of the derivative **6**, X-ray structural analyses of *A. suum* QFR and porcine SQR in complexes with the derivatives **5** and **6** were performed. The derivative **5** is bound to both enzymes in a similar manner as flutolanil: the phenyl group is situated in the same site as the flutolanil isopropoxybenzene ring and interacts with hydrophobic residues (*A. suum* QFR: Leu 60C and Trp 69C; porcine SQR: Ile 30C and Trp 35C). Further, in *A. suum* QFR the electrostatic interaction between the trifluoromethylbenzene ring and the guanidino group of Arg 76C is preserved (Figure 5a,b). In contrast, the conformation of the bound derivative **6** is markedly different from that of flutolanil in porcine SQR (Figure 5c), probably because the space between Trp 35C and Ile 30C cannot accommodate the larger pentafluorophenoxy group of the derivative **6**. The same might be true for *A. suum* QFR, although trials of the structure determination of the *A. suum* QFR-derivative **6** complex have not been successful yet.

### 2.4. Inhibitors with Higher Potency and Specificity

**Figure 6:**
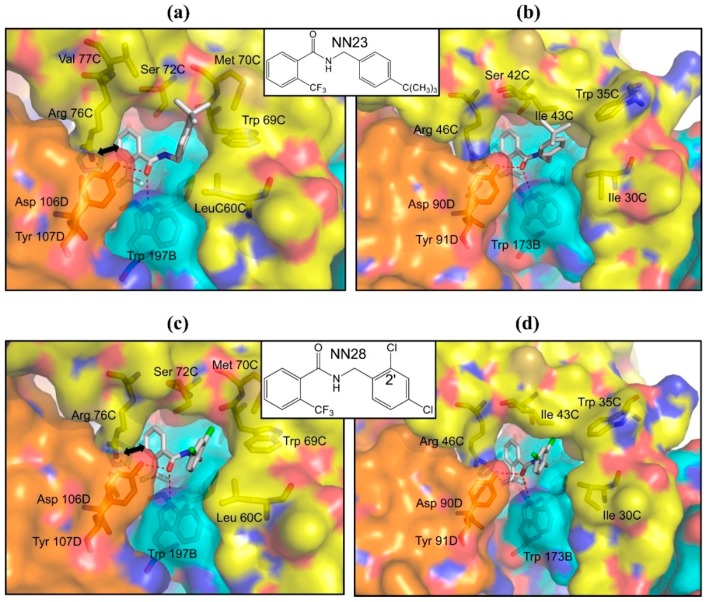
NN23 binding sites of (**a**) *A. suum* QFR and (**b**) porcine SQR; and NN28 binding site of (**c**) *A. suum* QFR. The trifluoromethyl F and carbonyl O atoms of NN23 and NN28 form hydrogen bonds with residues of *A. suum* QFR (Trp 197B, Arg 76C and Tyr 107D) and with those of porcine SQR (Trp 173B, Arg 46C and Tyr 91D) as indicated by red dotted lines. The bold bidirectional arrow in (**a**,**c**) shows an electrostatic interaction between NN23/NN28 and Arg 76C; (**d**) Model structure of the NN28 binding site speculated for porcine SQR. Possible hydrogen bonds are indicated by red dotted lines but the steric hindrance between the 2′-Cl atom of dichlorobenzene group and Ile 43C occurs in this model structure. Color codes are the same as Figure 4.

Flutolanil is composed of two aromatic rings that are linked by a peptide bond. Since it is highly restricted in its freedom of conformation due to the planar peptide linker, an investigation into the effect of a linker with higher rotational freedom on its potency was considered. Then we searched the compound library of Nihon Nohyaku Co., Ltd. (Tokyo, Japan) for flutolanil analogues possessing a –CO–NH–CH_2_– linker. An important criteria on the linker is the presence of a carbonyl group, this is because the carbonyl group accepts hydrogen bonds from Trp 197B and Tyr 107D in the *A. suum* QFR-flutolanil complex (Figure 4), and would be essential for the binding of analogues to the enzymes. Two very promising hits, NN23 and NN28, were found. The IC_50_ values of NN23 toward *A. suum* QFR and porcine SQR are 0.0055 and 111 μM, respectively, indicating that NN23 inhibits *A. suum* QFR about 20,000 times more strongly than porcine SQR. This is a significant improvement in both potency and specificity for *A. suum* QFR as compared with flutolanil. The structure of *A. suum* QFR in complex with NN23 (Figure 6a) reveals that the trifluoromethylbenzene ring, like flutolanil, interacts with the guanidino group of Arg 76C. In addition, hydrogen bonds of the carbonyl oxygen atom with Trp 197B and Tyr 107D are also preserved. On the other hand, the *tert*-butylphenyl group, similar to the isoprene side chain of the bound RQ (Figure 2a), occupies the channel between the quinone binding site and the exterior of the *A. sum* QFR molecule, making contacts with Leu 60C, Trp 69C, Met 70C and Val 77C within a distance of 4.0 Å. This conformation allows Leu 60C and Trp 69C to establish contact with each other as is observed in the RQ binding form (Figure 2a), which might release the possible energetically unfavorable strain caused by the binding of flutolanil. Although similar features are also observed in the structure of porcine SQR-NN23 complex (Figure 6b), the toxicity of NN23 toward porcine SQR is reduced to approximately half.

On the other hand, NN28 inhibits less effectively *A. suum* QFR (IC_50_ = 0.028 μM) and surprisingly it is virtually no longer effective against porcine SQR (IC_10_ > 90.0 μM). This explains why we were not able to prepare crystals of the porcine SQR-NN28 complex in spite of repeated trials. NN28 is bound to *A. suum* QFR in a similar manner to NN23 (Figure 6c); all interactions including electrostatic interaction between the trifluoromethylbenzene ring and the guanidino group of Arg 76C, hydrogen bonds to a fluorine atom of the trifluoromethyl substituent and the carbonyl group of the linker donated by Arg 76C, Trp 197B and Tyr 107D are observed. Conversely, the model structure of the porcine SQR-NN28 complex speculated from the experimentally determined structure of *A. suum* QFR-NN28 complex (Figure 6d) reveals that the chlorine atom at the 2′-position of the dichlorobenzene group collides with Ile 43C (1.79 Å). The covalent bond connecting the dichlorobenzene group and linker was rotated in order to avoid the dichlorobenzene group and Ile 43C collision, the result was a further collision of the chlorine atom with Trp 35C, Ile 30C and Trp 173B, one after another. Therefore, the model structure suggests that NN28 is unable to bind the porcine SQR.

## 3. Experimental Section

### 3.1. Purification

*A. suum* QFR was extracted and purified according to the previously described method [23] using reagents purchased from Wako Pure Chemical Industries, Ltd. (Osaka, Japan) unless otherwise noted. Briefly, a mitochondria-rich fraction was separated from minced muscle suspended in Chappell–Perry (CP) buffer (100 mM KCl, 50 mM Tris–HCl pH 7.4, 1 mM ATP, 5 mM MgSO_4_ and 1 mM EDTA) [36] by differential centrifugation. The enzyme was solubilized from the mitochondrial membrane suspended in buffer A (10 mM Tris–HCl pH 7.5, 1 mM sodium malonate) using 1.0% (*w*/*v*) sucrose monolaurate (SML) purchased from DOJINDO (Kumamoto, Japan) as a detergent. After incubating the mixture for 30 min at 277 K, the clear reddish-brown supernatant containing the solubilized enzyme was recovered by centrifugation for 1 h at 200,000× *g*. The purification was carried out by successive ion-exchange chromatography using DEAE Sepharose FF and Source 15Q (GE Healthcare, Uppsala, Sweden) columns. The enzyme was eluted with buffer A containing a linear gradient of 0–0.3 M NaCl in the presence of 0.1% (*w*/*v*) SML. Fractions containing pure *A. suum* QFR as judged by SDS-PAGE were pooled and the purified enzyme precipitated by adding solid PEG 3350 (HAMPTON RESEARCH, Aliso Viejo, CA, USA) to 0.15 g/mL was stored at 193 K.

The purification of porcine SQR from porcine heart mitochondria was carried out according to the method described by Huo *et al.* [37]. The method consists of extraction of the porcine heart mitochondrial membrane from fresh heart muscle by differential centrifugation, solubilization of SQR from the membrane using the detergent sodium cholate and the purification of SQR by ammonium sulfate fractionation.

### 3.2. Crystallization

Crystals of *A. suum* QFR were prepared by the microdialysis method [23] using a mixture of octaethyleneglycol monododecylether (C12E8) and *N*-dodecyl-β-d-maltoside (C12M) as a detergent. The purified enzyme, precipitated by PEG 3350 was dissolved in 10 mM Tris–HCl pH7.5 containing 1 mM sodium malonate, 0.6% (*w*/*v*) C12E8, 0.4% (*w*/*v*) C12M and 0.2 M NaCl. After incubation for 20 min on ice, the enzyme was precipitated by adding an equal volume of 40% (*w*/*v*) PEG 3350. The precipitate obtained by centrifugation was dissolved in the same buffer, incubated for 20 min on ice and mixed with an equal volume of 40% (*w*/*v*) PEG 3350 to precipitate the QFR. This procedure was repeated several times in order to replace SML with a detergent mixture containing C12E8 and C12M. The precipitate was finally dissolved in buffer A containing 0.06% (*w*/*v*) C12E8, 0.04% (*w*/*v*) C12M and 0.2 M NaCl, and then an equal volume of 23% (*w*/*v*) PEG 3350 was added to the QFR solution. After centrifugation to remove undissolved materials, the supernatant was sealed in a 5 μL microdialysis button and dialyzed against reservoir solution containing 15% (*w*/*v*) PEG 3350, 100 mM Tris–HCl pH 8.4, 200 mM NaCl, 1 mM sodium malonate, 0.06% (*w*/*v*) C12E8 and 0.04% (*w*/*v*) C12M. Dark red plate-shaped crystals with a maximum length of 100~200 μm were obtained in 2~3 days at 293 K.

Crystallization of porcine SQR was carried out according to the method described by Huo *et al.* [29]. The detergent C12M at a final concentration of 0.5% (*w*/*v*) was used for the crystallization by hanging drop vapor diffusion method. The enzyme dissolved in 25 mM HEPES (pH 7.2), 200 mM sucrose, 100 mM NaCl and 0.5 mM EDTA was crystallized using 25 mM HEPES (pH 7.2), 5% (*w*/*v*) PEG 4000, 3% (*w*/*v*) 1,6-hexanediol, 100 mM NaCl, and 10 mM CaCl_2_ as reservoir solution.

### 3.3. X-ray Diffraction Data Collection, Structure Determination and Refinement

X-ray diffraction data were collected on beamlines BL41XU (λ = 1.000 Å; Rayonix MX225HE CCD detector, Rayonix, Oak Avenue Evanston, IL, USA) and BL44XU (λ = 0.900 Å; Bruker-AXS SMART6500, Bruker-AXS, Yokohama, Japan) at SPring-8 (Harima, Japan), and on beamlines BL-17A (λ = 1.000 Å; ADSC Quantum 270r detector, ADSC, Stowe Drive Poway, CA, USA) and NW12A (λ = 1.000 Å; ADSC Quantum 210 detector, ADSC) at Photon Factory (Tsukuba, Japan) by the rotation method. For X-ray diffraction experiments at 100 K, a crystal mounted on a nylon loop was transferred to reservoir solution supplemented with 20% (*w*/*v*) glycerol and was then flash-frozen in liquid nitrogen stream. Data were processed and scaled using *HKL-2000* and *SCALEPACK* [38] (HKL Research Inc., Charlottesville, VA, USA).

The structure of the *A. suum* QFR in flutolanil-free form was solved by molecular replacement using the refined coordinates of porcine SQR (PDB code 1ZOY) as a search model [29]. *MOLREP* program [39] as implemented within *CCP4* [40] (http://www.ccp4.ac.uk/) was used for molecular replacement. Statistics of X-ray data collection and refinement are summarized in Table S1 and Table S2. Graphical representations were generated with PyMOL (http://www.pymol.org).

### 3.4. Enzyme Assays

The CP buffer containing the prepared mitochondrial fraction was replaced with 50 mM Tris–HCl buffer, pH 7.4 containing 1 mM disodium malonate, 210 mM mannitol, 10 mM sucrose, and 1 mM EDTA. Final concentrations of the *A. suum* and porcine mitochondria (quantified protein) were adjusted to 10 and 20 mg/mL, respectively. Mitochondrial fractions were separated in 50 μL aliquots, frozen by liquid nitrogen and stored at 193 K until use. Prior to assay, the mitochondrial suspensions were thawed on ice and SQR was reactivated by incubation at 298 K for 30 min in equal volume of 50 mM potassium phosphate buffer pH 7.4, containing 337 μM disodium malonate. SQR assay buffer composed of 50 mM potassium phosphate buffer pH 7.4, 0.1% SML, 2 mM potassium cyanide, and 60 μM of 2,3-dimethoxy-5-methyl-6-geranyl-1,4-benzoquinone (UQ2) was pre-equilibrated at 298 K for 20 min. The assay mixture was constituted by adding 15 μg (*A. suum*) or 30 μg (porcine) of reactivated mitochondrial fraction to the assay buffer and incubated for 3 min for background activity measurements. The SQR activity was initiated by the addition of 10 mM disodium succinate, and recorded as rate of UQ2 consumption, monitored at 278 nm (ε_278_ = 15 mM^−1^·cm^−1^) for three minutes in 1 mL quartz cuvettes with a Shimadzu UV-3000 dual wavelength spectrophotometer (Shimazu, Kyoto, Japan). Inhibition of SQR activities were assayed in the presence of varying concentration of inhibitors (diluted in DMSO) and the IC_50_s calculated as the concentration of inhibitor that caused 50% decrease in SQR activity relative to control (DMSO). No significant difference was observed between the IC_50_ of flutolanil determined for SQR and QFR activity of *A. suum* complex II. Hence, the more convenient SQR assay for *A. suum* complex II was used to determine the IC_50_ of flutolanil derivatives tested in this study.

## 4. Conclusions

In spite of the similarity in amino acid sequences and three-dimensional structures between *A. suum* QFR and porcine SQR, flutolanil specifically inhibits the enzymatic action of *A. suum* QFR. The structures of *A. suum* QFR and porcine SQR in flutolanil-free and flutolanil-bound forms revealed that flutolanil is bound to the quinone binding site of each enzyme in a rather different manner from that of quinone. This is probably due to the low conformational flexibility of flutolanil—it is composed of two aromatic trifluoromethylbenzene and isopropoxybenzene rings connected by a –CO–NH– linker. Although the binding of flutolanil to *A. suum* QFR and porcine SQR seems to cause energetically unfavorable strain in the enzymes, a close inspection of the structures suggests that this energy loss is compensated for by attractive interactions between flutolanil and the enzymes. The key interactions that appear to be responsible for the specificity and potency of flutolanil toward *A. suum* QFR were identified in the structure of *A. suum* QFR-flutolanil complex. The importance of these interactions was proved by structure-activity relationship studies with flutolanil derivatives. On the basis of this structural investigation, flutolanil analogues showing higher potency and specificity toward *A. suum* QFR than flutolanil were found. These inhibitors, NN23 and NN28, possess high conformational flexibility as a result of the –CO–NH–CH_2_– linker and are able to bind to *A. suum* QFR without causing the energetically unfavorable strain in the enzyme as shown by X-ray crystal structure analyses.

## Supplementary Information

**Table S1:** Data collection and refinement statistics.

Data Set	QFR-RQ	QFR-Flutolanil	QFR-Derivative 1	QFR-Derivative 2	QFR-Derivative 5	QFR-NN23	QFR-NN28
**Space Group**							
Space group	*P*2_1_2_1_2_1_	*P*2_1_2_1_2_1_	*P*2_1_2_1_2_1_	*P*2_1_2_1_2_1_	*P*2_1_2_1_2_1_	*P*2_1_2_1_2_1_	*P*2_1_2_1_2_1_
**Data Collection**							
*a*/*b*/*c* (Å)	122.8, 123.6, 219.8	124.3, 131.5, 222.5	123.9, 127.0, 219.3	122.8, 123.4, 219.0	123.7, 126.4, 220.9	124.2, 127.9, 220.5	124.1, 127.7, 220.7
X-ray source	SPring8 BL44XU	KEK-PF-AR NW12	KEK-PF-AR NW12	KEK-PF BL17A	SPring8 BL44XU	SPring8 BL44XU	SPring8 BL44XU
Wavelength (Å)	0.9000	1.000	1.0000	0.98000	0.9000	0.9000	0.9000
Temperature (K)	100	100	100	100	100	100	100
Resolution (Å)	50.0–2.75 (2.85–2.75) ^a^	50–2.9 (2.95–2.9) ^a^	50.0–3.3 (3.36–3.3) ^a^	50–3.65 (3.71–3.65) ^a^	50.00–3.4 (3.52–3.4) ^a^	50–2.25 (2.29–2.25) ^a^	50–3.1 (3.15–3.1) ^a^
Total reflections	499,461	328,842	283,779	196,929	261,990	907,709	326,789
Unique reflections	87,281	73,077	52,914	37,582	49,409	166,618	64,793
Completeness (%)	99.9 (100.0) ^a^	95.4 (87.2) ^a^	98.2 (93.7) ^a^	97.3 (94.1) ^a^	95.6 (93.0) ^a^	96.4 (91.6) ^a^	96.0 (95.7) ^a^
*R*_merge_ *I* ^b^	0.092 (0.759) ^a^	0.096 (0.624) ^a^	0.137 (0.449) ^a^	0.164 (0.575) ^a^	0.136 (0.390) ^a^	0.082 (0.566) ^a^	0.114 (0.561) ^a^
*I*/σ(*I*)	7.4 (2.2) ^a^	12.4 (1.3) ^a^	5.9 (3.3) ^a^	4.5 (2.4) ^a^	11.1 (2.7) ^a^	11.4 (2.9) ^a^	16.6 (3.0) ^a^
Redundancy	5.7 (5.4) ^a^	4.5 (3.0) ^a^	5.5 (4.7) ^a^	5.4 (4.6) ^a^	5.5 (3.1) ^a^	5.7 (4.8) ^a^	5.3 (4.7) ^a^
**Refinement**							
Resolution (Å)	20–2.75	40–2.91	30–3.3	20–3.66	20–3.4	20–2.25	20–3.1
No. of reflections	78,102	56,377	48,822	31,772	43,572	152,442	58,730
*R*_work_^c^/*R*_free_^d^	0.196/0.252	0.204/0.272	0.179/0.250	0.193/0.262	0.178/0.239	0.181/0.221	0.183/0.238
**Number of Atoms**							
Protein	17,889	17,862	17,978	17,956	17,967	18,007	17,978
FAD	106	106	106	106	106	106	106
Malonate (Fumalate)	14	16	14	16	14	14	14
FeS clusters	38	38	38	38	38	38	38
Heme	86	86	86	86	86	86	86
Inhibitor	44	46	40	40	50	48	44
Lipid	88	88	88	88	44	88	88
Solvent molecules	106	-	15	-	-	721	27
***B*-factors (Å^2^)**							
Protein	50.8	86.8	62.3	59.6	79.2	49.5	66.7
FAD	28.3	61.8	42.7	31.4	50.7	35.7	49.8
Malonate (Fumalate)	38.3	106.0	51.8	47.1	75.6	42.3	63.1
FeS clusters	31.8	59.5	45.8	33.2	49.1	35.8	51.2
Heme	56.5	75.7	70.1	73.8	89.5	47.7	70.6
Inhibitor	58.2	71.7	65.4	83.7	94.6	45.5	75.9
Lipid	71.1	119.7	110.4	-	100.6	72.4	91.7
Solvent molecules	30.0	-	17.3	-	-	44.6	43.5
**R.m.s. Deviation**							
Bond length (Å)	0.008	0.013	0.008	0.006	0.007	0.009	0.007
Bond angle (°)	1.35	1.60	1.41	1.14	1.27	1.45	1.23
PDB code	5C2T	3VRB	4YSZ	4YT0	4YTM	4YSX	4YSY

**Table S2:** Data collection and refinement statistics.

Data Set	SQR-Flutolanil	SQR-Derivative 1	SQR-Derivative 3	SQR-Derivative 5		SQR-Derivative 6			SQR-NN23
**Space Group**									
Space group	*P*2_1_2_1_2_1_	*P*2_1_2_1_2_1_	*P*2_1_2_1_2_1_	*P*2_1_2_1_2_1_		*P*2_1_2_1_2_1_			*P*2_1_2_1_2_1_
**Data Collection**									
*a*/*b*/*c* (Å)	70.4, 83.7, 292.6	71.2, 84.0, 293.7	71.5, 83.8, 295.0	71.7, 84.2, 294.4		72.6, 84.2, 295.6			71.4, 84.0, 294.8
X-ray source	SPring8 BL44XU	KEK-PF BL17A	SPring8 BL41XU	KEK-PF-AR NW12		SPring8 BL41XU			KEK-PF BL17A
Wavelength (Å)	0.9000	0.98000	1.0000	1.000		1.00000			0.9800
Temperature (K)	100	100	100	100		100			100
Resolution (Å)	50–3.0 (3.05–3.0) ^a^	50–3.6 (3.66–3.6) ^a^	50–3.39 (3.51–3.39)	50–3.2 (3.31–3.2) ^a^		50–3.3 (3.42–3.3) ^a^			50–3.1 (3.15–3.1) ^a^
Total reflections	184,860	123,966	172,365	139,252		109,564			142,584
Unique reflections	35,891	20,323	25,146	29,008		27,349			31,105
Completeness (%)	97.5 (95.1) ^a^	97.0 (77.8) ^a^	98.3 (93.9)	99.8 (100.0) ^a^		98.2 (99.4) ^a^			93.3 (94.9) ^a^
*R*_merge_ *I* ^b^	0.099 (0.464) ^a^	0.149 (0.553) ^a^	0.091 (0.594)	0.076 (0.277) ^a^		0.08 (0.46) ^a^			0.095 (0.516) ^a^
*I*/σ(*I*)	11.5 (2.8) ^a^	16.1 (2.2) ^a^	8.0 (3.2)	19.3 (5.2) ^a^		12.8 (2.0) ^a^			11.4 (1.7) ^a^
Redundancy	5.3 (4.4) ^a^	6.1(4.4) ^a^	3.7 (3.7)	4.8 (4.9) ^a^		4.0 (4.1) ^a^			3.9 (4.6) ^a^
**Refinement**									
Resolution (Å)	20–3.0	50–3.62	50–3.39		40–3.24		40–3.31	20–3.1	
No. of reflections	33,058	20,118	25,081		28,950		27,275	25,461	
*R*_work_^c^/*R*_free_^d^	0.206/0.258	0.256/0.305	0.236/0.286		0.203/0.253		0.257/0.307	0.208/0.266	
**Number of Atoms**									
Protein	8480	8480	8480		8480		8480	8480	
FAD	53	53	53		53		53	53	
Malonate (Fumalate)	–	–	7		7		7	–	
FeS clusters	19	19	19		19		19	19	
Heme	43	43	43		43		43	43	
Inhibitor	23	23	23		25		31	24	
Lipid	–	–	44		44		44	–	
Solvent molecules	–	–	–		–		–	22	
***B*-factors (Å^2^)**									
Protein	80.6	145.3	113.9		71.0		124.7	71.1	
FAD	70.9	127.5	84.5		56.1		100.3	58.4	
Malonate (Fumalate)	–	–	177.5		93.1		222.2	–	
FeS clusters	64.9	124.7	84.4		55.1		94.0	54.7	
Heme	75.1	135.0	95.7		52.5		102.4	59.9	
Inhibitor	84.1	138.0	104.1		61.7		137.5	62.7	
Lipid	–	–	147.8		88.9		153.4	–	
Solvent molecules	–	–	–		–		–	44.1	
**R.m.s. Deviation**									
Bond length (Å)	0.008	0.005	0.005		0.007		0.005	0.007	
Bond angle (°)	1.31	0.84	0.88		1.08		0.867	1.24	
PDB code	4YXD	3AE7	3AEA		3ABV		3AE9	4YTP	

^a^, Values in parentheses are for the highest resolution shell; ^b^, *R*_merge_ *I* = Σ*_h_* Σ*_i_*[|*I_i_*(*h*) − <*I*(*h*)>|/*Σ_h_ Σ_i_ I*(*h*)], where *I_i_* is the *i*th measurement and <*I*(*h*)> is the weighted mean of all measurements of *I*(*h*); ^c^, *R*_work_ = Σ*_h_*|*F*_o_ − *F_c_*|*/*Σ*_h_F*_o_, where *F*_o_ and *F*_c_ are the observed and calculated structure factor amplitudes of reflection *h*; ^d^, *R*_free_ is as *R*_work_, but calculated with 10% of randomly chosen reflections omitted from refinement.
